# Hypertriglyceridemia and atherosclerosis

**DOI:** 10.1186/s12944-017-0625-0

**Published:** 2017-12-06

**Authors:** Jia Peng, Fei Luo, Guiyun Ruan, Ran Peng, Xiangping Li

**Affiliations:** Department of Cardiovascular Medicine, The Second Xiangya Hospital, Central South University, 139 Middle Renmin Road, Changsha, Hunan 410011 China

**Keywords:** Triglyceride, Lipoprotein, Atherosclerosis, Atherosclerotic cardiovascular disease

## Abstract

Atherosclerotic cardiovascular disease (ASCVD) is the leading cause of death and it has been confirmed that increased low density lipoprotein cholesterol (LDL-C) is an independent risk factor for atherosclerosis. Recently, the increasing evidence has showed that hypertriglyceridemia is associated with incremental ASCVD risk. But the proatherogenic mechanism of triglyceride (TG) remains unclear. Therefore, this article focuses on the clinical studies and proatherogenic mechanism related to hypertriglyceridemia, in order to provide reference for the prevention and treatment of ASCVD.

## Background

Morbidity and mortality rates from ASCVD continue to be extremely high in the world. Hypercholesterolemia is known to be a major ASCVD risk factor and the LDL-C lowering therapy has attracted extensive attention which has become a cornerstone of primary and secondary prevention in ASCVD. However, the substantial residual risk of ASCVD often remains after adjustment for the certain risk factors such as LDL-C [1] or intensive LDL-C lowering with statins and other optimal therapies [2]. The Residual Risk Reduction Initiative (R3i) has previously highlighted atherogenic dyslipidaemia, defined as the imbalance between proatherogenic triglyceride-rich apolipoprotein B-containing-lipoproteins and anti-atherogenic apolipoprotein A1-lipoproteins (as in high density lipoprotein, HDL), as an important modifiable contributor to lipid-related residual cardiovascular risk [3]. Recently, evidences are accumulating to suggest that hypertriglyceridemia is causally associated with increased atherosclerosis risk [4–7]. This article will review relevant literature regarding the association between hypertriglyceridemia and atherosclerosis including clinical studies and the researches of proatherogenic mechanism.

## TG/TRLs and its atherogenic effects

### TG and TRLs

On account of the hydrophobic nature of TG, it must combine with associated proteins into lipoprotein particles that allow it to transport in the plasma [5]. TG is major component of triglyceride-rich lipoproteins (TRLs) which include chylomicrons (CM), very low density lipoprotein (VLDL) and their remnants created during metabolism of TG [5, 8].

Chylomicrons which are large apolipoprotein B48 (apoB48)-containing lipoproteins with a large TG core (80%–95%) [9], which are synthesized via enterocytes absorbing TGs from dietary fat and promoting the apoB48 [9] and enter the systemic circulation through the lymphatic system where they acquire apoC2, apoC3 and apoE.

VLDL particles incorporate the apolipoprtein B100 (apoB100) and the core of TG that are synthesized in hepatocytes from fatty acids and glycerol [10], and secrete into systemic circulation where they provide a source for energy for peripheral tissues. During the process of secretion, VLDL particles combine with apoC1, apoC2, apoC3 and apoE. Once in circulation, CM and VLDL can be hydrolyzed by lipoprotein lipase (LPL) along the luminal surface of capillaries, generating the production of free fatty acids and chylomicron remnants, and in succession progressively smaller VLDLs and intermediate density lipoproteins (IDLs), respectively [10].

LPL is the key enzyme for the metabolism of TRLs, bound to glycosylphatidylinositol high density lipoprotein binding protein 1 (GPIHBP1) which provides the platform to allow lipolysis to occur at the endothelial cell surface [11] and is synthesized by the capillary endothelial cells [12]. Remnants are generated when CM and VLDL particles are catabolized during TG hydrolysis by LPL and are concomitantly enriched in cholesterol esters by the action of the cholesterol ester transfer protein (CETP) [13].

And, microsomal triglyceride transfer protein (MTP) transfers TG from the cytosol to endoplasmic reticulum containing nascent apoB during the assembly of CM and VLDL in enterocytes and hepatocytes, respectively [14]. The reduction of MTP expression or after taking the MTP inhibitor can eliminate and inhibit its function, therefore reducing the biosynthesis and plasma level of both chylomicrons and VLDL, and consequently decreasing plasma levels of LDL and TG [15].

### Atherogenic effects of TRLs

A recent study has indicated that lipoproteins in the circulation normally flux into and out of the arterial wall by transcytosis by which lipoproteins can be transported across the endothelium [16]. Furthermore, the transcytotic transport system is restricted to lipoproteins smaller than approximately 70 nm in diameter, thereby excluding CM and larger VLDL particles [17]. However, their remnants can infiltrate into sub-endothelial space. In contrast to LDL, TRL remnants carry more cholesterol per particles than LDL, due to their larger size [18]. And each remnant particle contains approximately 40 times more cholesterol compared with LDL [19]. And, they do not need to be modified/oxidized to become atherogenic [20] and be taken up directly by macrophages. As discussed above, compared with LDL, TRL remnants may have a stronger atherogenic effect.

It was found that, in human and Watanabe heritable hyperlipidemic rabbits, apoB48- and apoB100-containing lipoproteins were detected in aortic intimal lesion implicated to promote atherosclerosis [21, 22]. And, it has been showed the presence of triglyceride-containing remnant lipoproteins in human atherosclerotic plaque [23] strongly indicated that TRLs take part in the development and progression of atherosclerotic lesion. Furthermore, it has been evidenced that elevated circulating levels of triglycerides in the non-fasting state, a marker for triglyceride (TG)-rich remnant particles, are associated with increased risk of premature cardiovasculardiseases [8, 24].

## The evidences of clinical studies

### Clinical observational studies and intervention trials

It has been believed that the residual risk of atherosclerosis after LDL-C lowering therapy is significantly associated with TRLs. A number of epidemiological or observational studies showed that fasting or non-fasting hypertriglyceridemia is a causal risk factor of cardiovascular diseases [6, 7] even in individuals who have already achieved guideline-recommended LDL-C target levels with lipid-lowering therapy [25]. The non-fasting triglyceride has gained more attention and investigation in patients with cardiovascular heart disease (CVD). Most people eat regularly throughout the day, therefore usually only fasting (defined as at least 8 h since the last meal) for a few hours before the breakfast, and non-fasting (defined as within 8 h since the last meal) TG concentrations might be a better indicator of average lipid concentrations in the blood rather than fasting concentrations. Moreover, postprandial elevations of hepatic and intestinal lipoproteins are evident in T2D patients, despite normal TG levels in the fasting state [26]. Furthermore, Langsted et al. has found that after normal food intake, individuals in the general population have a maximum mean change from fasting levels of +26 mg/dL for triglycerides at 1 to 4 h after the last meal [27]. And, the only modest increase in triglyceride levels during normal food intake, together with the recent demonstration of high predictive ability of non-fasting triglycerides for risk of cardiovascular events, suggest the possibility that non-fasting rather than fasting triglyceride levels could be used for cardiovascular risk prediction [27]. Additionally, these studies usually have the participants complete oral fat load test of 1 g of fat per 1 kg body weight and detect increases in triglycerides of 86.7 to 173.3 mg/dL [28]. However, most studies found that 30 g fat in a meal has no or very little effect on postprandial lipidemia including triglyceride levels [27]. In spite of this, since 2009, a non-fasting lipid testing (measured on a random blood sample irrespective of time since last meal) has become the clinical standard in Denmark [29], and another several clinical guidelines (e.g. in the UK, Europe, and Canada) including non-fasting lipid testing in the primary prevention setting have been released successively [26]. In 2016, Nordestgaard et al. supported to recommend flagging of abnormal concentrations of non-fasting triglycerides as ≥175 mg/dL, and this cut-point was optimal for cardiovascular risk prediction [30]. Non-fasting lipid measurement is a simple approach to assess postprandial lipids, however it does not allow for a complete functional assessment of postprandial lipid excursion and potential abnormalities in insulin resistant states [26]. Assessment of lipid parameters at fixed time points following ingestion of a high-fat meal (i.e. oral fat tolerance test (OFTT)) is a preferred methodology to ensure optimal comparability between test subjects. And, non-fasting lipid responding to fat-containing meals have been examined in research settings in human subjects for the past 30 years [31]. Nevertheless, OFTT methodology remains largely unstandardized. In addition, robust reference values, which are critical to interpret postprandial parameters, remain to be confirmed [26]. Thus, more studies are required to develop standard procedures and further determine the correlation between non-fasting triglycerides and the risk of CVD. In 2014, studies combining the Copenhagen City Heart Study and the Copenhagen General Population Study with about 100,000 individuals showed that high concentrations ≥495.6 mg/dL of non-fasting triglycerides were associated with high risk of ASCVD and all-cause mortality [20]. In addition, remnant cholesterol is the cholesterol content of triglyceride-rich lipoproteins, composed of VLDL and IDL in the fasting state and of these two lipoproteins together with chylomicron remnants in the non-fasting state. Elevated non-fasting plasma triglyceride is a marker of elevated non-fasting remnant cholesterol. A well-designed prospective study [24] of a total of 73,513 genotyped subjects from Copenhagen, of whom 11,984 had ischemic heart disease diagnosed between 1976 and 2010 to test the hypothesis that elevated non-fasting remnant cholesterol is a causal risk. In this study, non-fasting remnant cholesterol was calculated as non-fasting total cholesterol minus HDL-C minus LDL-C, and remnant cholesterol can be calculated directly from a standard lipid profile. Then, they illustrated that a non-fasting remnant cholesterol increase of 39 mg/dL is associated with a 2.8-fold causal risk for ischemic heart disease, independent of reduced HDL cholesterol. This implied elevated TRLs can cause CVD because the remnant cholesterol levels directly correlated with TRLs. Recently, Puri R et al. [32] analyzed the data from 9 clinical trials involving 4957 patients with coronary disease undergoing serial intravascular ultrasonography to measure coronary atheroma volume and investigated the relationship between achieved non-HDLC and TG levels with coronary atheroma progression regression rates in a large cohort of patients with established coronary disease. It was demonstrated that coronary atheroma progression overall was more closely tied with changes in non-HDLC than that in LDL-C and appeared to associate with TG levels only beyond 200 mg/dL, which supported a more prominent role for non-HDLC and TG lowering in combating residual cardiovascular risk.

However, the above observation studies remain some limitations, one of which is that risk factors typically are only measured once, therefore the observed association will only represent a single point estimate and will entail the problem of regression dilution bias. Another two major limitations are in the form of potential confounding and reverse causation. The randomized, double-blind trials by design can overcome the problem of confounding simply by the randomization method and avoid reverse causation to investigating that whether TG-lowering therapies can decrease the risk of ASCVD. The classical TG-lowering drugs containing niacin acid, fibrates and omega-3 fatty acids can effectively decrease the plasma levels of TG. Early clinical studies showed that fibrate therapy can decrease the risk of cardiovascular events, compared with placebo group [33]. Besides, during the FIELD study [34], a multinational, randomized controlled trial (RCT) with 9795 participants, some participants started other lipid-lowing therapy (statin), due to the findings of HPS trial [35]. And, the study showed that fenofibrate did not significantly reduce the risk of the primary outcome of coronary events (coronary heart disease death or nonfatal myocardial infarction) and total cardiovascular events. Because of the different rate of starting statin therapy, the higher rate of statin therapy in placebo group might have masked a moderately larger treatment benefit. However, it did not meet the standard of designing RCT. And then this question was addressed directly in the following ACCORD trial. Moreover, in study of niacin acid, HATS trial [36] suggested that niacin acid plus simvastatin therapy can reduce coronary atherosclerotic stenosis progression and significantly decrease the incidence of fatal cardiovascular events, nonfatal myocardial infarction and cardiac revascularization. Unfortunately, the results from two large clinical trials of AIM-HIGH [37] and HPS2-THRIVE [38] indicated that there was no incremental clinical benefit from the addition of niacin to statin therapy, and the combination therapy may increase the risk of side effects. And there were different findings in studies about the combination therapy with fenofibrate plus statin. ACCORD trial [39] involving 5518 subjects who accepted fenofibrate plus simvastatin or single simvastatin showed that the combination of fenofibrate and simvastatin did not reduce the rate of primary endpoints (fatal cardiovascular events, nonfatal myocardial infarction, or nonfatal stroke) compared with simvastatin monotherapy (2.2% vs. 2.4%, *P* = 0.32). But, there was nonsignificant heterogeneity in a subgroup, comparing patients who had a triglyceride level in the highest third (≥ 204 mg/dL) and an HDL-C level in the lowest third (≤ 34 mg/dL) with all the other patients (*P* = 0.057 for interaction). Furthermore, in this subgroup, the primary outcome rate was 12.4% in the fenofibrate group, versus 17.3% in the placebo group, whereas such rates were 10.1% in both study groups for all other patients, and they supported that there was a reduction of CVD risk in patients with elevated TG levels and decreased HDL-C. But, recent intervention studies and genetic studies strongly indicated that on a population level low HDL-C is not causally linked to atherosclerotic events low HDL-cholesterol and seems to be a good marker of atherosclerosis but not an appropriate target [40]. In addition, in 2014, Davidson MH et al. [41] designed FIRST trial which failed to demonstrate decreased carotid intima-media thickness progression and risk of CVD events with fenofibric acid plus atorvastatin compared with statin monotherapy in a higher risk patient population with hypertriglyceridemia (≥ 150 mg/dL). Some scholars thought the possible reasons of above negative results are that trials have excluded individuals with triglycerides >396 mg/dL and duration of treatment respectively. Therefore, it still needs more randomized controlled trials to confirm who can benefit from TG-lowering therapy or which treatment strategy is safe and effective (Table 1).

**Table 1:** Summary of clinical studies links TG/TRLPs with CVD

Study	Study design	Sample size	Study population	Baseline lipid level (mgl/dL)	Follow-up	Key findings
[25] Faerge-Man et al. 2009	meta-analysis (2 prospective, randomized, multicenter trials)	*N* = 15,779	with clinically evident coronary heart disease or a history of myocardial infarction	/	/	slightly increased TG levels are associated with higher risk of recurrence of CVEs in statin-treated patients
[24] Varbo et al. 2013	meta-analysis (3 studies)	*N* = 73,513	white subjects of Danish descent from Copenhagen, of whom 11,984 had ischemic heart disease	/	/	the elevated nonfasting remnant cholesterol and TRLPs that are causally related to increased risk for ischemic heart disease
[32] Puri et al. 2016	meta-analysis (9 clinical trials)	*N* = 4957	with coronary disease	/	/	Plaque progression overall was closely tied with changes in non-HDLC and appeared to associate with TG levels only beyond 200 mg/dL
[33] DAIS 2001	multicenter, double-blind, placebo-controlled, randomized trial	Fen (*N* = 207) Pla (*N* = 211)	with type 2 diabetes aged. 40–65 years, with or without previous coronary intervention	LDL(mean): 130.7(Fen) 132.6(Pla), HDL(mean): 39.1(Fen) 40.6(Pla), TG(median): 229.4(Fen) 214.3(Pla)	3 years	Fenofibrate could decreased significantly progression in minimum lumen diameter and percentage diameter stenosis (localised. coronary-artery disease)
[34] FIELD Study 2005	multicenter, Controlled, randomized trial	Fen (*N* = 4895, *n* = 944) Pla (*N* = 4900, *n* = 1776)	with type 2 diabetes	LDL(mean): 118.7(Fen)118.1(Pla),HDL(mean): 42.5(Fen)42.5(Pla), TG(median): 152.8(Fen) 151.9 (Pla)	5 years	Fenofibrate did not significantly reduce the risk of the primary outcome of coronary events but reduce total cardiovascular events
[36] HATS Study 2007	double-blind placebo-controlled with a two-by-two factorial design	N + S (*N* = 33) Antioxidant vitamins (*N* = 39) N-S + A (*N* = 40) all placebos (*N* = 34)	with clinical coronary disease and at least three stenoses of at least 30% of the luminal diameter or one stenosis of at least 50%	LDL:127(P)132(N + S)117(A)124(N-S + A), HDL: 32(P)31(N + S) 32(A)30(N-S + A), TG:203(P)202(N + S) 207(A)236(N-S + A)	3 years	Niacin plus Simvastatin provided marked clinical and angiographically measurable benefits in patiients with coronary disease and low HDL levels
[39] ACCORD Study 2010	multicenter, randomized trial	F + S (*N* = 2765) Pla + Sim (*N* = 2753) Subgroup: F + S (*N* = 485) Pla + Sim (*N* = 456)	with type 2 diabetes	LDL:100.0 ± 30.3(F + S) 101.1 ± 31.0(Pla), HDL:38.0 ± 7.8(F + S) 38.2 ± 7.8(Pla), TG(mean):164(F + S)160(Pla)	4.7 years	The use of combination fibrate–statin therapy,did not reduce cardiovascullar risk in the majority of patients with type 2 diabetes, rather than statin therapy alone, but can reduce the CVD risk in subgroup with elevated TG levels (≥204 mg/dL) and decreased HDL-C (≤ 34 mg/dL)
[37] AIM-HIGH Study 2011	randomized trial	N + S(or plus Eze) (*N* = 1718) Pla + Sim(or plus Eze) (*N* = 1696)	with 45 years of age or older and established cardiovascular disease	LDL(mean): 74.0 ± 22.7(Nia) 74.2 ± 23.4(Pla), HDL(mean): 34.9 ± 5.6(Nia) 34.5 ± 5.6(Pla), TG(median): 163(Nia)167.5 (Pla)	36 months	No incremental benefit of niacin in reducing cardiovascular events, despite significant increases in HDL-C levels and decreases in triglyceride levels
[38] HPS2-THRIVE Study 2013	multicenter randomized trial	ERN/LRPT (N = 12,838) Pla (*N* = 12,835)	with established cardiovascular disease	on the background of LDL-lowering therapy, LDL(mean):63.4, HDL(mean):44.1, TG (median): 126.7	3.9 years	adding ERN/LRPT to simvastatin 40 mg daily (or plus ezetimibe) increased the risk of myopathy
[41] FIRST Study 2014	multicenter, double-blind, placebo-controlled trial	FA135mg + Ato (*N* = 340) Pla + Ato (*N* = 342)	with mixed dyslipidemia and a history of coronary heart disease or risk equivalent	LDL(mean): 84.0(F+A)84.5(Pla),HDL(mean): 40.1(F+A)39.6(Pla),TG(median): 205.0(F+A) 193.0 (Pla)	104 weeks	FA plus Atorvastatin did not decrease cIMT progression in high-risk patients with mixed dyslipidemia and may achieve a clinical benefit in patients with TG level of ≥175 mg /dL

Abbreviations: *CVEs* cardiovascular events, *TRLPs* triglyceride-rich lipoproteins, *LDL* low-density lipoprotein*, HDL* high-density lipoprotein, *TG* triglyceride, *Fen* Fenofibrate, *Pla* Placebo, *FA* fenofibric acid, *Ato* Atorvastatin, *Nia* niacin, *Eze* ezetimib, *ERN/LRPT* Extended release niacin plus laropiprant, *F+A* fenofibric acid plus Atorvastatin, *F+S* Fenofibrate+Simvastatin, *N+S* Niacin+Simvastatin, *N-S+A* Niacin–Simvastatin+Antioxidants*, n* the sample size of starting other lipid-lowering therapy (statin) in both groups in FIELD study

According to the above studies or trials, we can get that the individuals assigned in control or experimental group, the baseline of TG did not exceed 1000 mg/dL. And, what is the link between severe hypertriglyceridemia and CVD? It has been found that moderately elevated plasma triglycerides signalize increased risk for cardiovascular diseases, and extremely elevated triglycerides (> 1000 mg/dL) signalize increased risk for pancreatitis [42]. Additionally, the severe hypertriglyceridemia mainly contains CM that leads to pancreatitis [43], and the hydrolysis of CM via LPL results in the production of free fatty acids and chylomicron remnants which can contribute to pancreatitis [44]. Whether the severe hypertriglyceridemia has none of association with atherosclerosis or CVD, it still further investigates the components of hypertriglyceridemia and mechanism which contributes to atherosclerosis.

Another attractive viewpoint is the nutraceuticals, such as red yeast rice, soybean proteins, plant sterols, soluble fibres and others, which contains significant lipid-lowering properties (decreasing level of LDL-C and TG or increasing level of HDL-C). A relatively large amount of epidemiological, clinical data and trials support the tolerability and safety of many nutraceuticals with demonstrated lipid-lowering action, including in patients intolerant to statins [45]. Moreover, a number of nutraceuticals have shown promising effects in terms of improving the lipid profile and modifying cardiovascular risk [46]. Furthermore, the recent 2016 European guidelines for dyslipidemia management consider the possibility to use some lipid-lowering nutraceuticals [47]. In general, a large number of nutraceuticals have been tested in available trials, demonstrating their lipid-lowering effects, and some clinical trials have reported that many nutraceuticals have an additive effect to lipid-lowering drugs, allowing the statin doses to be reduced without diminishing the results in terms of LDL-C and TG reduction. It is, however, important to emphasize that nutraceuticals cannot replace lipid-lowering therapy but may essentially help to optimize it (reducing cardiovascular residual risk) [48]. But, it has to be clearly stressed that there are still no outcome studies proving that nutraceuticals can prevent CVD morbidity or mortality [48, 49].

### Genetic studies and Mendelian randomization studies

In the genetic evidence [50], the fact that monogenic disorders of TG metabolism, such hyperlipoproteinemia type 3,edispose individuals to CVD suggested that raised TG and remnant cholesterol levels contribute to the process of atherosclerosis [51]. Then, a recent Mendelian randomization study based on data from 10,208 individuals included in the Copenhagen City Heart Study found that subjects with genetically confirmed reduction in non-fasting plasma TG levels had reduced all-cause mortality [4]. Furthermore, Do R et al. [5] approved that the strength of a variant effect on TG levels strongly correlated with the magnitude of its effects on coronary artery disease, even after adjustment for effects on LDL-C and HDL-C through meta-analysis involving 188,578 genotyped individuals with 185 different single nucleotide polymorphisms. Furthermore, Genome-wide association studies (GWAS) have implicated numerous novel genes in TG metabolism and coronary atherosclerotic diseases (CAD) pathogenesis in humans, and the discovery of rare variants in TG genes and the subsequent association of these variants with CAD has strengthened both mechanistic research and drug discovery relevant to lipidemic diseases [52]. Additionally, sequence variants in several key genes involved in the metabolism of TRLs, such as those encoding LPL and the proteins regulating it, appear to be strongly associated with CVD risk [4, 24]. These results supported that the genes involved in encoding key components of the TRLs and metabolism of TRLs are strongly associated with CVD risk.

More recently, several Mendelian randomization studies has been conducted to evaluate whether factors involved in TRL metabolism are causally associated with atherosclerosis and coronary heart disease (CHD), and provide robust evidence for TG contributing to ASCVD. Mendelian randomization studies are based on the segregation and independent assortment of specific genotypes according to the laws of Mendelian genetics. And, Mendelian randomization studies of human genetics have many similarities with randomized, double-blind trials, which are not affected by confounding factors seen in observational epidemiologic studies and thus advantages over observational studies in conventional epidemiology. Several Mendelian randomization studies conducted using data from Copenhagen City Heart Study found that genetic variants in LPL resulted in reduced triglyceride levels, and that a higher number of triglyceride-decreasing LPL alleles was associated with increased survival [4]. Moreover, a Mendelian randomization meta-analysis was conducted using data from 17 studies involving 62,199 participants and 12,099 CHD events which suggested that triglycerides, both the unrestricted allele score based on 67 single-nucleotide polymorphisms (SNPs) and the restricted allele score based on 27 SNPs were significantly associated with CHD [53].

Taken together, the genetic data for triglycerides and TRLs align with the epidemiologic and clinical data, and support a causative role for these lipids in ASCVD. Nevertheless, we still need evidences from randomized intervention trials showing that lowering of TG and remnant cholesterol can reduce the major adverse cardiovascular events. Fortunately, such studies have already started: in individuals already receiving a statin, add-on placebo-controlled, triglyceride-lowering omega-3 fatty acids therapy to reduce residual risk is currently being tested in 2 ongoing trials. (REDUCE-IT: NCT01492361, STRENGTH: NCT02104817). These studies will provide valuable information on the utility of omega-3 fatty acids in combination with statin therapy in high-risk patients with TG levels to guide the prevention and treatment of ASCVD.

## The possible mechanism of TRLs atherogenicity

**Fig. 1:**
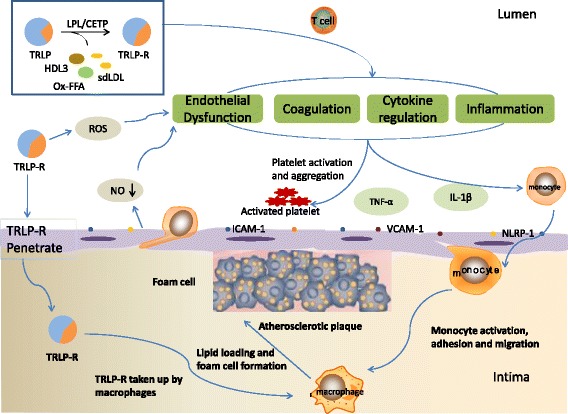
The possible mechanisms of TRLs in the process of the onset and progression of atherosclerosis. TRLs and its lypolitic products hydrolysed by LPL and CETP, containing TRL remnants (TRL-R), sd-LDL, HDL3 (HDL remodeling), oxidized free fatty acids (ox-FFA) and others, can increase the production of reactive oxygen species (ROS) and decrease nitric oxide (NO) released by endothelium and upregulate the endothelial expression of some molecules (ICAM-1, VCAM-1 and NLRP-1), which promote endothelial dysfunction. And, TRLs and its products penetrate in intima and induce inflammation contributing to monocyte activation, adhesion and migration. Meanwhile, the endometrial leukocytes can take up TG or cholesterol contents of TRL-R to form the foam cells, and then develop into core of atherosclerotic plaque. Additionally, a number of cytokines (containing TNF-α, IL-1β and others) and T cells take part in process of atherosclerosis and the whole process of atherosclerosis involves in platelet activation and aggregation to induce a procoagulant state and clot formation, in hypertriglyceridemia. **Abbreviations**: **LPL** lipoprotein lipase **CETP** cholesterol ester transporter protein**TRL** triglyceride-rich lipoproteins **TRL-R** triglyceride-rich lipoprotein remnants **sdLDL** small and dense LDL **HDL** high-density lipoprotein **ICAM-1** intercelluar adhesion molecule-1 **VCAM-1** vascular cell adhesion molecule-1 **NLRP-1** nucleotide-binding domain-like receptor family pyrin domain-containing protein 1 **TNF-α** tumor necrosis factor-α **IL-1β** interleukin-1 β

The mechanism of TRLs atherogenicity has attracted much attention, but it is still not very clear. The recent researches supported that TRLs are easy to get deposited on the wall of artery, which may damage the endothelium and enter into the arterial intima via defect of endothelium, the location of the atherosclerotic plaque, and enhance recruitment and attachment of monocytes to induce generation of foam cell. At the same time, TRLs take part in the development and progression of atherosclerosis by stimulating inflammation and regulating various cytokines (Fig. 1).

### TRLs and endothelial dysfunction

The dysfunction of endothelium has been demonstrated to precede the formation of atherosclerotic lesion and is one of the first steps involved in the pathophysiology of atherosclerosis. TRL remnants have been suggested to promote endothelial dysfunction, which potentiates atherogenesis [54]. It has been shown that the flow-mediated or acetylcholine-induced vasodilatation is associated with nitric oxide (NO) released by endothelium, which is one of sensitive index of endothelium-dependent vasodilation. Clinical studies found that the postprandial rapid rise in serum TG levels after a high-fat meal was significantly related to endothelial dysfunction via evaluating the impairment in flow-mediated vasodilatation [55]. It has also been shown that the remnant lipoprotein contributes to the impairment of endothelium-dependent vasomotor function in human coronary arteries [56]. In 2016, Lucero D et al. [57] analyzed the effect of circulating isolated TRLs from subjects on endothelial function in 40 patients with metabolic syndrome by means of the in vitro assay from which dose-response curves, and highlighted that the strong tendency to a positive correlation between triglyceride content in TRLs and the grade of inhibition of acetylcholine-mediated vasorelaxation exerted by TRLs. Almost at the same time, another study [58] involving in 4887 subjects who were enrolled in the flow-mediated vasodilation (FMD)-Japan registry was designed to investigate cross-sectional associations between serum triglyceride levels and endothelial function assessed by measurement of FMD of brachial artery. Then they found that, serum triglyceride levels of more than 98.4 mg/dL were independently associated with the low quartile of FMD (less than 3.9%) after adjustment for age, sex, and cardiovascular risk factors, including HDL-C which suggested that triglycerides are an independent predictor of endothelial function.

The similar results were also observed in animal experiments. Matsumoto S et al. [59] used the postprandial hypertriglyceridemic rabbits (PHT rabbits) as a new dyslipidemic model showing remarkably high levels of serum TG after feeding standard rabbit chow with little increase in serum cholesterol and healthy Japanese white rabbits (JW rabbits) as control group, investigating the link between postprandial elevated TG levels and endothelial dysfunction in the development of atherosclerosis [59]. They found that JW rabbits (12-month-old, 35-month-old) did not show atherosclerotic lesion, while hypertriglyceridemic rabbits (12-month-old PHT rabbits) showed significant intimal thickening in aorta. In this study, it was showed that the endothelial function in PHT rabbits was diminished by the acetylcholine-induced vascular relaxation, which is probably due to the decreased production of NO. The results demonstrated that hypertriglyceridemia can damage endothelial function and take part in the process of the onset and progression of atherosclerosis.

In addition, it has been established that TRL remnants can increase the production of reactive oxygen species (ROS), which may increase vascular endothelial permeability, and that high concentration ROS can cause cellular injury and death, in particular the endothelial cells [60]. Remnant-like lipoprotein particles may impair endothelial function by direct and indirect effects on nitric oxide synthase [61]. The imbalance of reactive ROS and nitric oxide may promote endothelial dysfunction, and lead to cardiovascular complication [62], particularly, in hypertriglyceridemia [63].

Finally, TRLs may suppress the atheroprotective and anti-inflammatory effects of HDL [64]_,_ which have been shown to significantly correlate with impairment of endothelium-dependent coronary vasodilation.

### TRLs and foam cells

It has been proved that activated macrophages, which incorporate oxidized/ modified lipoproteins and transform into lipid-rich foam cells, are abundant in the atherosclerotic lesions [65]. Triglyceride accumulation in macrophages, which is related to macrophage oxidative stress, was shown to further increase the mitochondrial generation of reactive oxygen species, promoting foam cell formation [66]. Moreover, VLDL particles from patients with hypertriglyceridemia are rich in apoE, which can lead to a conformational change in the VLDL particles that facilitate binding to the macrophage scavenger receptor. CM remnants and IDL are also small enough to enter the subendothelial space where they are taken up in an unregulated fashion by scavenger receptors on macrophages, leading to foam cell formation [15]. The lipoprotein particles such as oxygenized LDL (oxLDL) and TRLs bind to the scavenger receptor on macrophages and unregulated uptake of the modified lipoprotein particle causes macrophage accumulation of lipids, which is the formation process of a foamy cytoplasm and term foam cells [15]. Furthermore, it has been found that CM remnants contribute to atherosclerosis by migrating to the subendothelial space, where they induce leukocyte activation and promote foam cell formation similar to oxLDL [67], and induce monocyte activation and enhance monocyte and postprandial neutrophils migration [68].

### TRLs and inflammation

A number of the available literature has consistently indicated inflammation to be an essential risk factor for the onset and progression of atherosclerosis. The accumulation of postprandial TRLs led to the retention of remnants particles in the arterial wall [17] and stimulated an inflammatory response and oxidative stress [17, 69]. It has been discovered that TRLs participate in the inflammation via direct and indirect ways. A high concentration of lipolytic products from LPL-mediated TRLs hydrolysis, such as oxidized free fatty acids, along with TRLs themselves, are deemed to activate a number of proinflammation and proapoptotic signaling pathways that play a fundamental role in the pathogenesis of atherosclerosis [18]. Multiple studies have suggested that the oxidized free fatty acids can increase the expression of inflammatory interleukins and cytokines, leading to endothelial inflammation [60, 69, 70] while TRL remnants have been shown to upregulate the endothelial expression of intercelluar adhesion molecule-1 (ICAM-1) and vascular cell adhesion molecule-1 (VCAM-1), facilitating the transendothelial migration of leukocytes to sites of inflammation and enhancing inflammatory response [71]. Recently, Bleda S et al. [72] attempted to observe the nucleotide-binding domain-like receptor family pyrin domain-containing protein 1 (NLRP1) inflammasome gene expression in human arterial endothelial cells (HAECs) exposed to plasma with the elevated levels of TG and VLDL-C. They found that TG and VLDL contribute to plaque rupture and arterial inflammation through various mechanisms, one of which is NLRP1 inflammasome pathway, and the NLRP1 inflammasome activation triggered by TG and VLDL-C may represent a significant source of inflammation in endothelial cells.

TRLs can also impact HDL levels and its particle size. At the setting of the higher TG levels, the greater exchange between TG of apoB-containing lipoproteins and cholesterol ester (CE) of HDL via CETP results in the TG-rich and CE-poor HDL particles which can be catabolized faster and more rapidly than large and CE-rich HDL, and the process brings the consequence with lower levels of HDL-C. And, a recent study also revealed that high postprandial triglyceridemia induced a shift of HDL size towards large particles, and cholesterol depletion with TG enrichment of HDL3 subclasses [73]. The change of HDL structure is related with their antioxidant capacity that may be affected by HDL remodeling [74]. In addition, the current view maintains that distinct HDL particle subpopulations composed of unique clusters of specific HDL associated proteins perform specific biological functions, especially paraoxonase 1(PON1), an athero-protective protein, which show improved anti- oxidative, anti-inflammatory and lipid cargo carrying functions [75]. Whether the TG-rich and CE-poor HDL particles contain different content of PON1 or the change of PON1 activity, by which contribute to atherosclerosis. However, a study found that PON-1 activity did not decrease and significantly increase along the postprandial stage [73]. And, the further studies will undoubtedly need to be conducted to investigate the roll of such structural modifications (other components or proteins, i.e. apo AI or myeloperoxidase and platelet-activating factor acetylhydrolase,) on other HDL anti-atherogenic functions as a result of sustained postprandial lipemia and the mechanism TG enrichment of HDL3 subclasses contribute to atherosclerosis. It has been discovered that HDL carries apoB-bound sphingosine-1-phosphate (S1P), a lipid mediator with anti-inflammatory properties, which promotes the development of inflammatory T helper 1 cells while suppressing differentiation of Treg cells [76]. Thus, the higher TG levels can alter HDL concentration and size into the lower level of large TG-rich HDL which cannot participate in the process of anti-inflammation via T regulatory cells; on the contrary, these can contribute to inflammation via proinflammatory T cells [77].

In another side, mild-moderate hypertriglyceridemia (TG levels between 200 and 800 mg/dL) are associated with low levels of HDL-C, small and dense LDL (sd-LDL) particles, atherogenic TG-rich remnants [78]. And, sd-LDL is generated during the delipidation process by hepatic lipase (HL) from VLDL1 (TG-rich lipoprotein) to IDL and LDL particles and a number of clinical studies strongly suggest that a predominance of sd-LDL is associated with CVD risk, in hypertriglyceridemia [79] [80]. The possible mechanisms have been proposed for the atherogenic potency of sd-LDL [80]. Because of the small size and a lower affinity for LDL receptors than LDL, sd-LDL particles penetrate easily into the arterial wall and cannot be easily cleared from plasma, respectively. Together with, sd-LDL has a high affinity for proteoglycans in the arterial wall, which results in a prolonged residence time in the subendothelial space where it can contribute to the lipid storage and atherosclerosis plaque development. Moreover, sd-LDL particles contain less anti-oxidative vitamins (vitamin E) and are therefore more susceptible to oxidation than larger forms of lipoproteins. Lastly, the other possible mechanisms should be further study, for example, sd-LDL may induce the stimulation of plasminogen-activator-inhibitor 1 and accelerate thromboxane A2 synthesis [80].

Additionally, it has also been shown TRLs or TRL remnants can induce early monocyte and neutrophil activation resulting in inflammation [81, 82].

### TRLs and regulation of cytokines

A number of cytokines are involved in the onset and progression of atherosclerosis. TRL remnants have been shown to induce endothelial cell apoptosis via increased secretion of the proapoptotic cytokines, tumor necrosis factor-α (TNF-α), and interleukin-1 β (IL-1β), a process which can contribute to vascular injury and atherosclerosis [83]. It has been demonstrated that TNF-α has a substantial effect on endothelial cell dysfunction and is one of the most important molecules in cellular inflammation, regulating the expression of nitric oxide synthase (NOS) and thereby influencing the production of NO which is involved in endothelial dysfunction [84]. Studies have also suggested that TNF-α concentrations are positively associated with VLDL-C concentrations [84], which also can be found in the PTH rabbits [59]. A recent study has showed TNF-α overexpression increased expression of JAM-1, which promoted the chemotaxis and exudation of cells to cause atherosclerosis [85].

It is known that adipocytes can produce adipocytokines such as adiponectin with anti-atherogenic and anti-inflammation effects and leptin with lipolytic and appetite suppressing effects [86]. Moreover, a recent study has reported that there was a low adiponectin levels and the adiponectin mRNA was downregulated in PTH rabbits [59].

It has also been found that TRLs and their remnants can induce a procoagulant state, enhance platelet aggregation and clot formation, and amplify the coagulation cascade by following two ways: First, increasing the levels of fibrinogen, factors VII and XII; Second, upregulating the expression of plasminogen activator inhibitor-1 and plasminogen activator inhibitor-1 antigen [87].

In brief, a large number of studies have shown that hypertriglyceridemia contributes to the development and progression of atherosclerosis. The proatherogenic mechanism of TRLs seems rather complicated and needs to be further explored. Based on current knowledge and the evidence of clinical studies, controlling and lowering plasm TG levels is one of the important measures to further reduce the residual risk of CVD events in ASCVD patients or at high risk for ASCVD after achieving guideline-recommended LDL-C target levels. We also expect more clinical trials designed to support above view.

## Abbreviations

apoB100: Apolipoprtein B100
apoB48: Apolipoprotein B48
ASCVD: Atherosclerotic cardiovascular disease
CAD: Coronary atherosclerotic diseases
CE: Cholesterol ester
CETP: Cholesterol ester transfer protein
CHD: Coronary heart disease
CM: Chylomicron
CVD: Cardiovascular heart disease
FMD: Flow-mediated vasodilation
GPIHBP1: Glycosylphatidylinositol high density lipoprotein binding protein 1
GWAS: Genome-wide association studies
HAECs: Human arterial endothelial cells
HDL: High density lipoprotein
HL: Hepatic lipase
ICAM-1: Intercelluar adhesion molecule-1
IDL: Intermediate density lipoprotein
IL-1β: Interleukin-1 β
JW rabbits: Japanese white rabbits
LDL-C: Low density lipoprotein cholesterol
LPL: Lipoprotein lipase
MTP: Microsomal triglyceride transfer protein
NLRP1: Nucleotide-binding domain-like receptor family pyrin domain-containing protein 1
NO: Nitric oxide
NOS: Nitric oxide synthase
OFTT: Oral fat tolerance test
oxLDL: Oxygenized LDL
PHT Rabbits: postprandial hypertriglyceridemic rabbits
PON1: Paraoxonase 1
ROS: Reactive oxygen species
sd-LDL: Small and dense LDL
SNPs: Single-nucleotide polymorphisms
TG: Triglyceride
TNF-α: Tumor necrosis factor-α
TRLs: Triglyceride-rich lipoproteins
VCAM-1: Vascular cell adhesion molecule-1
VLDL: Very low density lipoprotein
